# Litter Dynamics and Nutrient Stocks in a Chronosequence of Hyperxerophilous Forest Under the Effect of Clear Felling

**DOI:** 10.1002/ece3.72100

**Published:** 2025-10-09

**Authors:** Renisson Neponuceno de Araújo Filho, Maria Betânia Galvão dos Santos Freire, Fernando José Freire, Rinaldo Luiz Caraciolo Ferreira, Ludmilla Morais Pereira, Luiz Diego Vidal Santos

**Affiliations:** ^1^ Department of Rural Technology Federal Rural University of Pernambuco Recife Pernambuco Brazil; ^2^ Department of Agronomy Federal Rural University of Pernambuco Recife Pernambuco Brazil; ^3^ Department of Forest Science Federal Rural University of Pernambuco Recife Pernambuco Brazil; ^4^ Graduate Program in Forest Engineering Santa Catarina State University Lages Santa Catarina Brazil; ^5^ Department of Human Sciences and Philosophy State University of Feira de Santana Feira de Santana Bahia Brazil

**Keywords:** biogeochemical cycle, clusters, decomposition of litter, forest management, multivariate analysis

## Abstract

Clear‐cutting disrupts forest structure, alters litter accumulation, and impairs nutrient cycling in dry forest ecosystems. This study evaluated the effects of clear‐cutting on litter and nutrient stocks in hyperxerophilous Caatinga in northeastern Brazil in areas under different regeneration stages (0, 6, 9, 12, 25, 50 years, and a reference site) in northeastern Brazil. Stocks and concentrations of total carbon (TC), total nitrogen (TN), phosphorus (P), potassium (K), calcium (Ca), magnesium (Mg), and sulfur (S) were measured. Statistical analyses included Generalized Linear Models (GLM) with gamma distribution, MANOVA for multivariate comparisons, Principal Component Analysis (PCA) to identify structural gradients, and hierarchical cluster analysis to classify lignocellulosic profiles. Results showed that clear‐cutting substantially reduces litter and nutrient stocks, particularly N and P, and increases the TC/TN ratios—especially in the litter—negatively affecting litter accumulation. After 50 years, regeneration had not restored pre‐disturbance levels. Litter biomass was functionally associated with soil P, Ca, and S. PCA revealed two main gradients: one axis of recalcitrance (related to FDA, cellulose, and NDF) and one axis of lignin‐cellulose complexity, which discriminated materials with different decomposition potentials. Multivariate analyses revealed gradients linked to recalcitrance and litter decomposition potential. These findings indicate that the TC/TN ratio and nutrient availability regulate litter persistence and ecosystem resilience. Enrichment planting with species having lower TC/TN ratios, green manuring, and biodiversity restoration are recommended to improve litter quality, accelerate nutrient cycling, and promote recovery of ecosystem functions in drylands.

## Graphical abstract

We investigated litter accumulation and nutrient stocks across a forest regeneration chronosequence in hyperxerophilous Caatinga following clear‐cutting. Using multivariate statistics and structural decomposition indices, we identified functional litter profiles and their association with nutrient cycling and soil resilience. Our findings highlight how biomass quality and regeneration time jointly regulate ecosystem recovery in semi‐arid tropical forests.

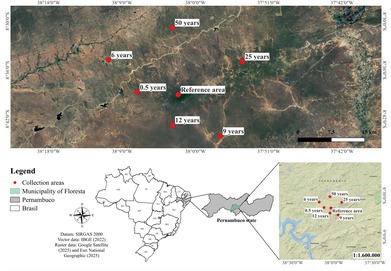

## 1 Introduction

The Caatinga is a forest located in the semi‐arid Northeast of Brazil, occupying an area of around one million square kilometers, covered by deciduous vegetation (Queiroz et al. 2017). These formations have different physiognomies according to the time of year (Medeiros et al. 2022). In the rainy season, greenery takes over the landscape, while in the dry season most of the plants lose their leaves in response to the scarcity of water (Ramos et al. 2023).

Around 40% of the Caatinga's original area is still covered by native vegetation, mostly composed of secondary formations in different stages of regeneration, a direct result of the degradation caused by practices such as clear‐cutting for firewood production and the opening up of agricultural areas under the system of shifting agriculture (Noutcheu et al. 2024). This pattern of intensive land use has been the main factor behind forest reduction and the expansion of secondary vegetation in the biome (Araujo et al. 2023).

In addition to clear‐cutting, extensive areas of Caatinga are subjected to intensive native grazing, where predominantly herbaceous vegetation is consumed during the rainy season and shrub and tree leaves are used in the dry season (Formiga et al. 2020; da Cunha et al. 2022). This continuous use of plant biomass triggers critical soil degradation processes, including a reduction in litter stocks (Jing et al. 2022), the export of nutrients such as nitrogen, phosphorus, and potassium via biomass consumption and removal, and surface soil compaction, which can compromise water infiltration into the soil (Leul et al. 2023).

Despite the recognized influence of this type of management on the degradation of ecosystem services, there are still significant gaps in the understanding of the effects of continuous biomass removal on the biogeochemical cycles and functional resilience of the Caatinga, especially in the different post‐disturbance successional stages (Silva et al. 2024).

Forests make nutrients available to the soil mainly through the deposition and decomposition of litter (Li et al. 2024). This process is fundamental for the cycling of elements such as nitrogen and carbon, as well as providing energy and substrate for maintaining the life of terrestrial macro and microfauna.

Recent studies highlight that litter not only nourishes the soil, but also sustains the biological and microbial activity responsible for releasing and transforming these nutrients (Giweta 2020; Azad et al. 2020). In forest ecosystems, litter plays a central role in nutrient cycling processes (Pradhan and Behera 2020), acting in the supply and replacement of essential elements, in the formation and maintenance of soil organic matter (SOM) (Sultana et al. 2021), as well as contributing to the conservation of biodiversity and the support of various ecosystem functions.

Furthermore, the quantity and quality of litter production provide valuable information on the dynamics of nutrient cycling in forest ecosystems, and are directly influenced by the senescence and phenology of species. As Ulbricht et al. (2025) and Zhou et al. (2021) have shown, the amount of senescent biomass that reaches the ground is related to leaf retention time and the stage of tree development. Furthermore, species such as deciduous trees show greater leaf deposition in specific periods, influencing the composition and volume of litter and, consequently, nutrient cycling (Sheshnitsan and Sheshnitsan 2024).

The breakdown of forest structure due to natural disturbance or anthropogenic actions alters (Chen, Jomaa, et al. 2024) ecosystem processes. In addition to the interruption in the absorption of elements by plants, other processes such as evapotranspiration, decomposition, and the transformation of elements through the nutrient cycling process are altered (Campbell et al. 2022). Clear‐cutting is the main disturbance in these forests, as intensive management for timber production can affect the distribution and flows of nutrients in forest ecosystems (Chazdon 2014).

Areas that have been more altered over time have a high number of opportunistic species that grow quickly, have a short life cycle, and are resistant to abiotic factors in the environment, showing greater efficiency in the production of plant biomass in a shorter space of time (Giweta 2020). Late secondary and dominant species are slower growing and prefer shadier environments, that is, they are sensitive to high light intensities; drought and clear‐cutting have been shown to decrease the biomass of these species (Matsuo et al. 2024). Along these lines, differences in litter production between nearby stretches may be linked to the different degrees of disturbance found within the same forest type (Palviainen et al. 2005).

Studies on litter dynamics in the Caatinga are still scarce globally (Kirmse et al. 1987; Tiessen et al. 1992; Alves et al. 2017; Holanda et al. 2017). This gap limits the understanding of nutrient cycling and post‐disturbance recovery processes in this unique ecosystem. Specifically, the study aimed to answer the following questions: (1) How does the time elapsed since clear‐cutting affect the quantity and quality of accumulated litter? (2) How do nutrient stocks in the litter (TC, TN, P, K, Ca, Mg, S) vary along the forest regeneration chronosequence? (3) How are structural characteristics of the litter related to nutrient retention and decomposition potential at different successional stages? The hypothesis is that longer regeneration times result in greater litter accumulation and higher nutrient stocks due to increased biomass input, and that areas in early stages of regeneration present litter with lower nutritional quality (higher TC/TN ratio and lignin content), reflecting slower decomposition rates and reduced nutrient cycling efficiency. Therefore, investigating litter production and its relationship with forest regeneration at different successional stages is fundamental to quantifying edaphic resilience and subsidizing conservation strategies. In this context, the aim of this study was to determine the effect of clear‐cutting on accumulated litter and nutrient stocks in areas of Caatinga subjected to different forest management times.

## 2 Materials and Methods

### 2.1 Study Area Description

**FIGURE 1:**
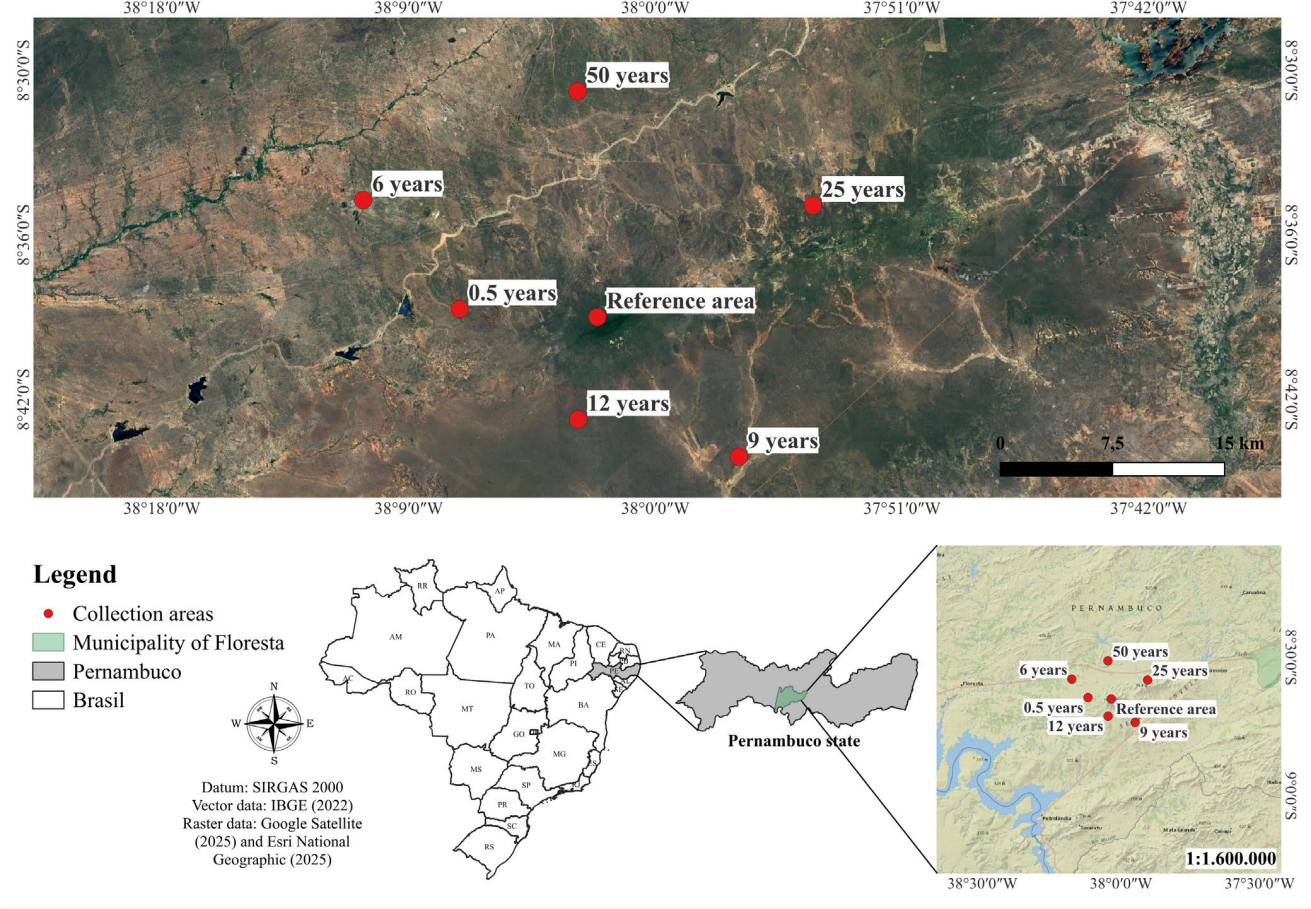
Geographic location and litter sampling points of the study area in the municipality of Floresta, Pernambuco State, Brazil.

The study was carried out in areas of hyperxerophilous Caatinga in the municipality of Floresta, state of Pernambuco, Brazil (08°30′ S; 37°57′ W), under a BSh climate (hot and dry semi‐arid) according to Köppen‐Geiger (Koppen 1948). The average annual temperature is 28°C, the average annual rainfall is 500 mm (concentrated between November and March), and the potential evapotranspiration reaches 1646 mm per year. The terrain is flat to gently undulating (Figure 1).

**FIGURE 2:**
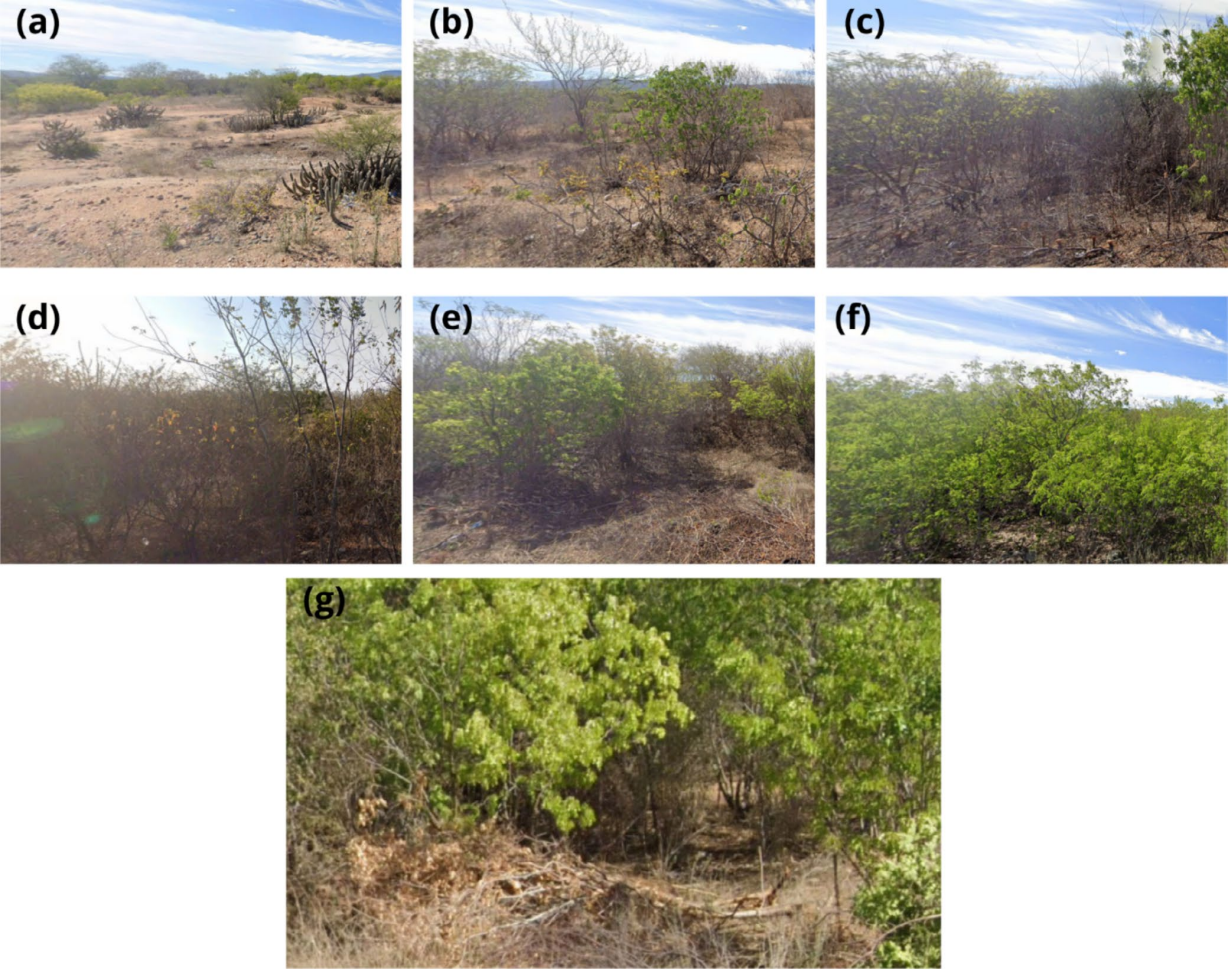
Caatinga areas under different stages of natural regeneration where litterfall samples were collected. (a) 0.5 years; (b) 6 years; (c) 9 years; (d) 12 years; (e) 25 years; (f) 50 years; (g) Reference area (no recent cutting history).

Seven areas were selected with different times of natural regeneration after clear‐cutting, making up a chronosequence with ages of 0.5, 6, 9, 12, 25, and 50 years, as well as a reference area (R) with no recent history of anthropogenic disturbance, with over 80 years of regeneration (Figure 2). The age of each regeneration area was determined based on official records from a Sustainable Forest Management Plan issued by the Pernambuco State Environmental Agency (CPRH 2008), ensuring reliable historical information on the time since clear‐cutting.

Litterfall samples were collected in October 2014 across all regeneration stages. To ensure that the litterfall stock data collected in 2014 still adequately represent the current conditions of the study area, a comparative land use and land cover analysis was conducted using MapBiomas maps from 2015 to 2023, the most recent year available.

**FIGURE 3:**
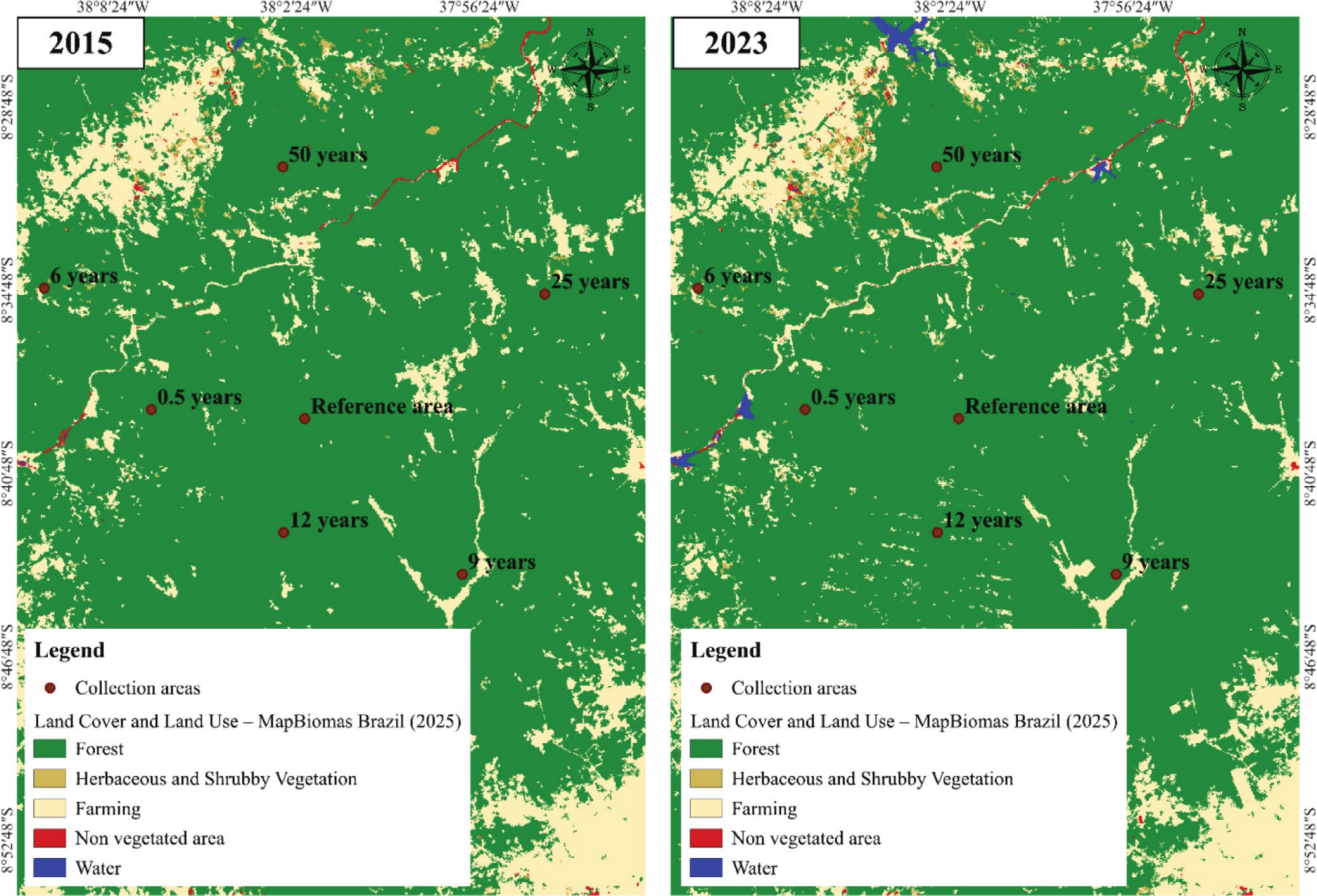
Land cover and land use—Comparison between 2015 and 2023.

This analysis allowed for the verification of possible landscape changes and confirmed the stability of the area over the period (Figure 3).

### 2.2 Reference Area

The reference area (08°36.423′ S; 37°59.290′ W) has 80 ha and preserved vegetation. The vegetation is dominated by *Cenostigma bracteosum* (Tul.) Gagnon and G.P. Lewis (catingueira—30.34%), *Mimosa ophthalmocentra* Mart. ex Benth (jurema—26.51%), *Croton rhamnifolius* H. B. K. (velame—7.05%), *Manihot carthaginensis* subsp. *glaziovii* (Müll. Arg.) Allem. (maniçoba—6.27%) and *Jatropha mollissima* (Pohl) Baill. (pinhão‐bravo—4.98%).

The soil in the area was classified as Inceptisols (Soil Taxonomy 2014), with undulating relief and very stony conditions. The Ap horizon (0–20 cm) shows a 5YR 4/6 (moist) color, weak subangular blocky structure, very hard consistency when dry, friable when moist, and plastic and sticky when wet, with a clear and smooth transition to the subsurface horizon. The texture is loam, with a soil density of 1.37 g cm^−3^ and total porosity of 47.91%. The pH is slightly acidic (6.62), with high base saturation (85%), base sum of 8.95 cmolc kg^−1^, exchangeable sodium percentage of 2.28%, potential cation exchange capacity of 10.53 cmolc kg^−1^, and total organic carbon of 14.61 g kg^−1^.

The Bi horizon (20–50 cm) presents a 5YR 4/4 (moist) color, rock‐like structure, with no development of granular aggregates, showing direct occurrence of bedrock. The texture is sandy clay loam, with a pH of 6.68, total organic carbon of 10.96 g kg^−1^, base sum of 9.31 cmolc kg^−1^, potential cation exchange capacity of 10.53 cmolc kg^−1^, base saturation of 88%, and exchangeable sodium percentage of 3.13%.

### 2.3 50 Years

The 50‐year‐old area (08°30.970′ S; 37°59.025′ W) has 60 ha. *Cenostigma bracteosum* (catingueira—34.3%), *Mimosa ophthalmocentra* (jurema—11.9%), *Aspidosperma pyrifolium* Mart. (pereiro—6.4%), *Cnidoscolus bahianus* (Ule) Pax and K.Hoffm. (favela‐mansa—5.6%) and *Anadenanthera colubrina* (Vell.) Brenan (angico—5.3%) are the dominant species.

The soil in the area was classified as Inceptisols (Soil Taxonomy 2014), with gently undulating relief, stony condition, and imperfect drainage. The Ap horizon (0–10 cm) has a moist color of 7.5YR 3/4, subangular blocky structure, very friable consistency when moist, sandy clay loam texture, soil density of 1.51 g cm^−3^, and total porosity of 45.09%. The pH is neutral (7.10), with high base saturation (84%), base sum of 9.44 cmolc kg^−1^, exchangeable sodium at 0.80%, potential cation exchange capacity of 11.22 cmolc kg^−1^, and total organic carbon of 14.98 g kg^−1^.

The Bi horizon (10–30 cm) presents a moist color of 2.5YR 5/4, subangular blocky structure, friable consistency when moist, sandy clay loam texture, soil density of 1.46 g cm^−3^, and total porosity of 45.11%. The pH remains neutral (7.04), base saturation is high (89%), base sum is 8.71 cmolc kg^−1^, exchangeable sodium is 3.42%, potential cation exchange capacity is 9.95 cmolc kg^−1^, and total organic carbon is 10.30 g kg^−1^.

### 2.4 25 Years

The 25‐year‐old area (08°33.416′ S; 37°56.548′ W; 60 ha) was clear‐cut and subsequently abandoned. The vegetation is dominated by *Cenostigma bracteosum* (catingueira—37.1%), *Mimosa ophthalmocentra* (jurema—21.1%), *Jatropha mollissima* (pinhão‐bravo—8.9%), *Pityrocarpa moniliformis* (angico‐de‐bezerro—5.3%) and *Thiloa glaucocarpa* (cipaúba‐de‐boi—2.4%).

The soil in the area was classified as Inceptisols (Soil Taxonomy 2014), with flat relief and non‐stony condition. The A horizon (0–20 cm) has a moist color of 5YR 4/4, medium subangular blocky structure, slightly hard consistency, very friable when moist, and plastic and sticky when wet. The texture is sandy loam, with a soil density of 1.42 g cm^−3^ and total porosity of 45.59%. The pH is moderately acidic (5.65); base saturation is eutrophic (72%), with a base sum of 6.17 cmolc kg^−1^, exchangeable sodium at 1.05%, potential cation exchange capacity of 8.55 cmolc kg^−1^, and total organic carbon of 17.16 g kg^−1^.

The Bi horizon (20–120+ cm) also has a moist color of 5YR 4/4, medium subangular blocky structure, slightly hard consistency, friable when moist, and plastic and sticky when wet. The texture is also sandy loam, with a soil density of 1.51 g cm^−3^ and total porosity of 42.37%. The pH is strongly acidic (4.72), base saturation remains eutrophic (74%), with a base sum of 6.06 cmolc kg^−1^, exchangeable sodium at 1.96%, potential cation exchange capacity of 8.16 cmolc kg^−1^, and total organic carbon of 12.01 g kg^−1^.

The four areas with recent regeneration followed selective cutting protocols with the exclusion of protected species, as *Myracrodruon urundeuva* (M. Allemão) Engl. (aroeira), *Schinopsis brasiliensis* Engl. (baraúna), *Spondias tuberosa* Arruda (umbuzeiro), *Commiphora leptophloeos* (Mart.) J.B.Gillett. (imburana), *Erythroxylum* spp., and conservation of individuals with a diameter of less than 2 cm and riparian vegetation. Regeneration occurs through regrowth and natural germination.

### 2.5 12 Years

In the 12‐year‐old area (08°35.940′ S; 37°59.409′ W; 90 ha), the vegetation is composed of *Cenostigma bracteosum* (catingueira—25.7%), *Mimosa ophthalmocentra* (jurema—11.01%), *Manihot carthaginensis* subsp. *glaziovii* (maniçoba—9.29%), *Croton rhamnifolius* (velame—8.08%) and *Myracrodruon urundeuva* (aroeira—7.41%).

The soil in the area was classified as Alfisols (Soil Taxonomy 2014), with gently undulating relief, non‐stony condition, imperfect drainage, subangular blocky structure, and friable consistency when moist. The A horizon (0–30 cm) presents a 10YR 4/3 (moist) color, sandy loam texture, soil density of 1.71 g cm^−3^, total porosity of 36.43%, moderately acidic pH (5.70), eutrophic base saturation (69%), base sum of 6.90 cmolc kg^−1^, exchangeable sodium of 0.60%, potential cation exchange capacity of 9.93 cmolc kg^−1^, and total organic carbon of 17.85 g kg^−1^.

The E horizon (30–80 cm) has a 10YR 5/3 (moist) color, sandy loam texture, soil density of 1.73 g cm^−3^, total porosity of 35.21%, slightly acidic pH (6.16), eutrophic base saturation (79%), base sum of 7.02 cmolc kg^−1^, exchangeable sodium of 1.02%, potential cation exchange capacity of 8.85 cmolc kg^−1^, and total organic carbon of 13.82 g kg^−1^. The 2Bt horizon (80–100+ cm) presents a 10YR 5/2 (moist) color, with a clear and flat transition to sandy clay loam texture, showing an increase in clay content (29.63%), soil density of 1.80 g cm^−3^, total porosity of 32.84%, near‐neutral pH (6.71), eutrophic base saturation (82%), base sum of 6.87 cmolc kg^−1^, exchangeable sodium of 3.71%, potential cation exchange capacity of 8.36 cmolc kg^−1^, and total organic carbon of 9.68 g kg^−1^.

### 2.6 9 Years

In the 9‐year‐old area (08°35.485′ S; 37°59.351′ W; 90 ha), the most abundant species are *Cenostigma bracteosum* (catingueira—29.7%), *Mimosa ophthalmocentra* (jurema—8.31%), *Pityrocarpa moniliformis* (angico‐de‐bezerro—8.1%), *Croton rhamnifolius* (velame—7.3%) and *Myracrodruon urundeuva* (aroeira—6.02%).

The soil in the area was classified as Alfisols (Soil Taxonomy 2014), with gently undulating relief, slightly stony condition, imperfect drainage, subangular blocky structure, and friable consistency when moist. The A horizon (0–15 cm) has a 10YR 5/6 (moist) color, sandy loam texture, soil density of 1.70 g cm^−3^ and total porosity of 36.32%, moderately acidic pH (5.27), eutrophic base saturation (63%), base sum of 5.80 cmolc kg^−1^, low exchangeable sodium content (1.19%), potential cation exchange capacity of 9.21 cmolc kg^−1^, and total organic carbon of 15.30 g kg^−1^.

The E horizon (15–60 cm) maintains a 10YR 5/6 (moist) color, sandy loam texture, soil density of 1.72 g cm^−3^, total porosity of 35.58%, moderately acidic pH (5.39), eutrophic base saturation (69%), base sum of 6.38 cmolc kg^−1^, exchangeable sodium of 1.53%, potential cation exchange capacity of 9.18 cmolc kg^−1^, and total organic carbon of 12.85 g kg^−1^. The 2Bt horizon (60–75+ cm) presents a 10YR 5/2 (moist) color, with a clear and smooth transition to a sandy clay loam texture due to an increase in clay content (29.65%), soil density of 1.79 g cm^−3^, porosity of 33.46%, consistently moderately acidic pH (5.28), eutrophic base saturation (72%), base sum of 6.57 cmolc kg^−1^, exchangeable sodium of 4.74%, potential cation exchange capacity of 9.08 cmolc kg^−1^, and total organic carbon of 7.79 g kg^−1^.

### 2.7 6 Years

The 6‐year‐old area (08°34.665′ S; 38°00.910′ W; 90 ha) has *Cenostigma bracteosum* (catingueira—36.31%), *Mimosa ophthalmocentra* (jurema—10.69%), *Jatropha mollissima* (pinhão‐bravo—9.7%), *Myracrodruon urundeuva* (aroeira—5.08%) and *Aspidosperma pyrifolium* (pereiro—5.02%) as the dominant species.

The soil in the area was classified as Inceptisols (Soil Taxonomy 2014), with gently undulating relief and slightly stony condition. The A horizon (0–15 cm) has a 10YR 4/4 (moist) color, medium subangular blocky structure, slightly hard consistency, friable when moist, and plastic and sticky when wet. The texture is sandy loam, with a soil density of 1.40 g cm^−3^ and total porosity of 46.56%. The pH is moderately acidic (4.95), with eutrophic base saturation (51%), a base sum of 4.23 cmolc kg^−1^, low exchangeable sodium content (1.70%), potential cation exchange capacity of 8.22 cmolc kg^−1^, and total organic carbon content of 10.74 g kg^−1^.

The Bi horizon (15–80+ cm) has a 10YR 4/6 (moist) color, medium subangular blocky structure, slightly hard consistency, friable when moist, and plastic and sticky when wet. The texture remains sandy loam, with a soil density of 1.53 g cm^−3^ and total porosity of 41.82%. The pH is slightly acidic (5.83), with eutrophic base saturation (61%), a base sum of 5.48 cmolc kg^−1^, exchangeable sodium content of 4.68%, potential cation exchange capacity of 8.98 cmolc kg^−1^, and total organic carbon content of 7.04 g kg^−1^.

### 2.8 0.5 Years

The 0.5‐year‐old area (08°35.518′ S; 37°59.741′ W; 90 ha) was managed six months before sampling and is dominated by *Cenostigma bracteosum* (catingueira—29.7%), *Mimosa ophthalmocentra* (jurema—20.1%), *Myracrodruon urundeuva* (aroeira—10.69%), *Pityrocarpa moniliformis* (angico‐de‐bezerro—10.1%) and *Jatropha mollissima* (pinhão‐bravo—8.31%).

The soil in the area was classified as an Alfisol according to Soil Taxonomy (2014), featuring gently undulating relief, slightly stony condition, imperfect drainage, subangular blocky structure, and friable consistency when moist. The A horizon (0–5 cm) has a sandy loam texture, color 2.5YR 5/4 (moist), soil density of 1.71 g cm^−3^, and total porosity of 36.19%. It is moderately acidic (pH 5.63), with a dystrophic base saturation (46%), base sum of 3.81 cmolc kg^−1^, potential cation exchange capacity of 8.27 cmolc kg^−1^, low exchangeable sodium (2.66%), and total organic carbon of 11.57 g kg^−1^. The E horizon (5–35 cm) maintains a sandy loam texture and the same color (2.5YR 5/4, moist), with a soil density of 1.74 g cm^−3^, porosity of 34.83%, pH 5.52, eutrophic base saturation (51%), base sum of 4.50 cmolc kg^−1^, potential cation exchange capacity of 8.82 cmolc kg^−1^, and total organic carbon of 10.38 g kg^−1^.

The 2Bt horizon (35–50+ cm) shows an increase in clay content (29.07%), with a clear, smooth transition to a sandy clay loam texture, color 2.5YR 5/3 (moist), soil density of 1.77 g cm^−3^, porosity of 34.20%, pH 5.67, eutrophic base saturation (62%), base sum of 4.95 cmolc kg^−1^, potential cation exchange capacity of 7.95 cmolc kg^−1^, and total organic carbon of 7.40 g kg^−1^. This is the most fertile horizon in the profile, despite having an exchangeable sodium content of 5.53%. The profile shows a clear differentiation of horizons and evidence of clay illuviation, typical features of Alfisols.

The floristic composition of the areas is complemented by other species typical of the Caatinga, such as *Bumelia sertorum* Mart. (quixabeira), *Pithecellobium dumosum* Benth. (jurema‐branca), *Cassia* spp. (pau‐besouro), *Bauhinia aculeata* L. (mororó), *Pithecellobium* spp. (feijão‐bravo), *Sapium* spp. (burra‐leiteira) and *Rosmarinus officinalis* L. (rosemary grown in borders).

### 2.9 Litter Sampling, Processing, and Chemical Analysis

**FIGURE 4:**
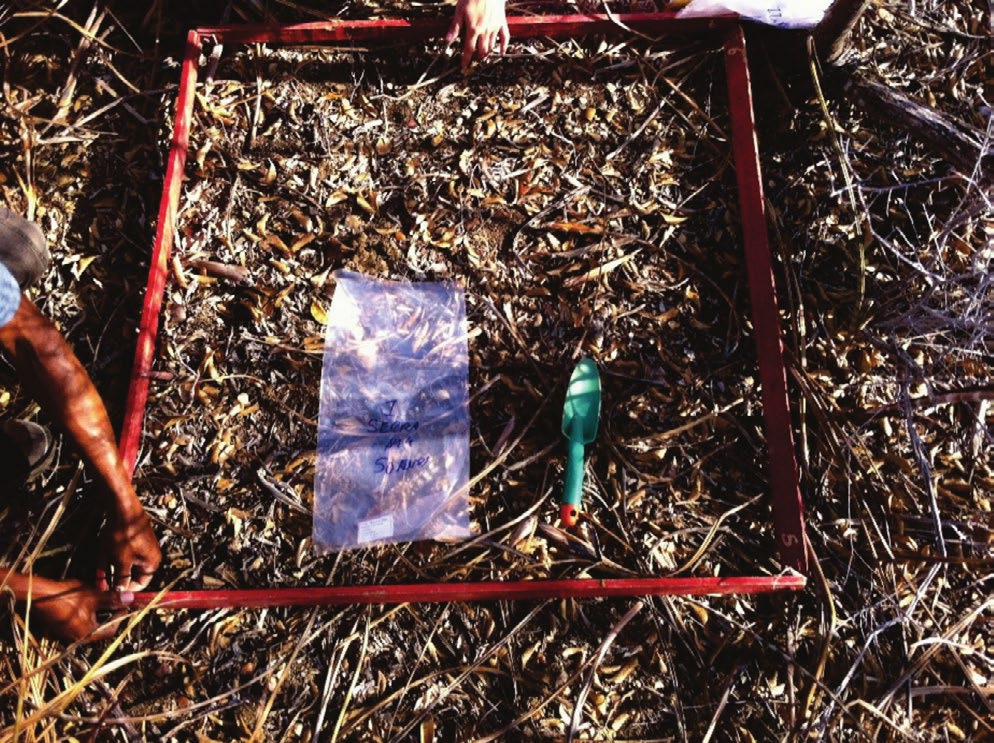
Litter sampling in the study area in the municipality of Floresta, Pernambuco State, Brazil.

Collections to determine the litter stock on the forest floor were carried out in October 2014, using a commonly used collector called a template, made of wood with dimensions of 1.0 m^2^ × 0.30 m in height. In addition, layers of partially decomposed litter samples were collected. In each area, 103 random samples were collected within a radius of 2.5 m from the base of each tree (Figure 4).

The material collected was sorted manually and separated into three fractions: leaves, branches up to 0.03 m in diameter, and miscellany. The material that could not be identified consisted of plant and animal remains and fecal material. The fractions were packed in paper bags and dried in a forced circulation oven at 65°C until they reached a constant weight. The samples were then weighed to obtain the dry mass of the plant components.

After drying, the fractions were ground (< 0.5 mm) in a laboratory and analyzed for the available forms of phosphorus (as H_2_PO_4_^−^), potassium (as K^+^), calcium (as Ca^2+^), and magnesium (as Mg^2+^). Determinations were made using aqueous extracts obtained by nitroperchloric digestion: phosphorus by spectrophotometry, potassium by flame photometry, and calcium and magnesium by atomic absorption spectrometry (Teixeira et al. 2017). Total carbon (TC), total nitrogen (TN) and sulfur (S) were quantified by dry combustion (CHNS/O) in a Perkin Elmer PE‐2400 Series II elemental analyzer. The partially decomposed litter was sieved at 5 mm, and the pH was determined at a ratio of 1:10 (soil: water), according to Blakemore et al. (1987). The acid detergent fiber (FDA) and acid detergent lignin (ADL) fractions were obtained using the Ankom Filter Bag Technique (Van Soest 1963, 1967); cellulose was estimated as CEL = FDA–ADL, and lignin was corrected by incineration in a muffle furnace (550°C/4 h). Nutrient stocks were calculated by multiplying the biomass input by the concentration of each element.

### 2.10 Statistical Analysis

The data was submitted to multivariate analysis of variance using Multivariate Generalized Linear Models (Multivariate GLM), with bootstrap resampling (*n* = 1000), with the aim of simultaneously evaluating the effect of topographic position and soil depth on different functional sets of edaphic attributes (Friedl and Stadlober 1997).

The statistical analyses adopted multivariate Generalized Linear Models (GLM), rather than traditional ANOVA, due to the presence of multiple intercorrelated dependent variables and the greater flexibility of GLM in terms of specifying the distribution of residuals, making it more robust in contexts with small or heteroscedastic samples.

The choice of GLM with gamma distribution for the models was based on three main criteria. Firstly, the nature of the dependent variables, litter and nutrient stocks (TC, TN, P, K, Ca, Mg, S), which showed an asymmetric distribution, with asymmetry coefficients greater than 1.5 and clear patterns of heteroscedasticity in the residues, which made the assumptions of normality unfeasible, as indicated by the Shapiro–Wilk test (*p* < 0.01).

Secondly, the comparison between alternative distributions showed that the gamma distribution performed better than the normal and log‐normal distributions, with differences of more than 12.7 units in the Akaike Information Criterion (AIC) (Akaike 1974) and more than 10.3 in the Bayesian Information Criterion (BIC), according to the methodology of McCullagh and Nelder (2019).

In addition, the adequacy of the model was diagnosed using multiple metrics: the Deviance/gl (0.069) and Pearson/gl (0.051) ratios remained below 1, demonstrating conformity with the assumed distribution.

Cameron and Trivedi (1990)'s dispersion test did not indicate overdispersion (*p* = 0.321), and the studentized residuals remained within 2.5 standard deviations. The identity link function was maintained after evaluating the linearity between predictors and response via component‐plus‐residual plots, which revealed no significant non‐linear patterns (*p* > 0.05 in the lack of fit test), ensuring the adequacy of the model's parameterization.

As for the multivariate variance tests, the homogeneity of the covariance matrices between the groups was confirmed by the Box M test, and multivariate normality was considered plausible based on the distribution of the residuals and the robustness conferred by the resampling procedure. Main effects and interactions were tested using Pillai, Wilks, Hotelling, and Roy multivariate statistics.

For significant effects, multiple comparisons were made between pairs of means with Bonferroni correction in order to control for type I error. The adjusted means were estimated based on marginal effects, and comparisons between groups were made with Bonferroni correction. In addition, independent linear models were fitted for each depth in order to investigate the intra‐stratified variation in attributes as a function of topographic position. All statistical analyses were carried out in the R environment (R Core Team 2024).

Principal Component Analysis (PCA) was used to identify latent gradients of variability between the attributes NDF, hemicellulose, lignin, cellulose, and FDA. The variables were previously standardized (*z*‐score) to ensure comparability between different scales. The selection of principal components followed the Kaiser criterion, retaining those with eigenvalues greater than 1. Factor loadings with a modulus equal to or greater than 0.40 (| ≥ 0.40|) were considered relevant for structural interpretation (Jolliffe 2014). The results were visualized using two‐dimensional biplots of the first two principal components (PC1 and PC2), containing 95% confidence ellipses for the groupings. The analyses were conducted in the RStudio environment (R Core Team 2024), using the FactoMineR, factoextra, and ggplot2 packages.

To complement the multivariate exploratory analysis, a Hierarchical Cluster Analysis (HCA) was applied based on the Euclidean distances between the standardized samples. The linkage method adopted was Ward. D2, which minimizes intra‐group variance at each merging iteration (Randriamihamison et al. 2021). The optimum number of clusters was defined based on visual inspection of the dendrogram and the stability of the groupings.

Average structural patterns per group were identified based on the average lignin, cellulose, and NDF content in each cluster. In addition, a structural recalcitrance index was calculated based on the ratio between lignin and cellulose as a synthetic metric of relative resistance to decomposition. The clusters were visualized using a dendrogram with chromatic coding and annotation of the groups formed. All the steps were implemented in RStudio.

To identify linear patterns between accumulated litter biomass (PeS) and soil chemical attributes, simple linear regression models (ordinary least squares) were fitted for each combination of litter fraction (Leaves, Twigs, Miscellaneous and Total) and predictor variable (P, K, Ca, S, TN, TC/TN). The regressions were implemented individually per pair of variables, and the results were expressed graphically with fitted lines via geom_smooth(method = “lm”; ggplot2). The coefficient of determination (*R* ^2^) and the *p*‐value of each model were displayed in the respective facets. Models with *p* < 0.05 were considered significant.

## 3 Results

### 3.1 Total Litter

The adjusted GLM model indicated a statistically significant effect of the “Environment” factor on accumulated litter stocks (PeS) [*χ* ^2^ Wald (6) = 1135.90; *p* < 0.001], with a good fit according to the model's quality criteria (Deviance/df = 0.069; Pearson/df = 0.051).

The adjusted marginal estimates showed higher accumulations in areas R and 50 years, whose averages differed significantly from all the other environments (*p* < 0.001). Area R had the highest average litter stocks (Exp(B) = 2709.62 kg ha^−1^; 95% CI: 2348.92–3070.32), followed by area 50 years (Exp(B) = 1436.89 kg ha^−1^; 95% CI [1020.30–1853.47]; *p* < 0.001).

The areas with the greatest tree cover, such as the reference area and the area with 50 years of regeneration, showed the highest biomass accumulation values, while environments with younger and more open vegetation, such as the 0‐year‐old area, recorded the lowest average adjusted values (Exp(B) = 2366.16 kg ha^−1^; 95% CI [2053.79–2788.21]; *p* < 0.001).

**FIGURE 5:**
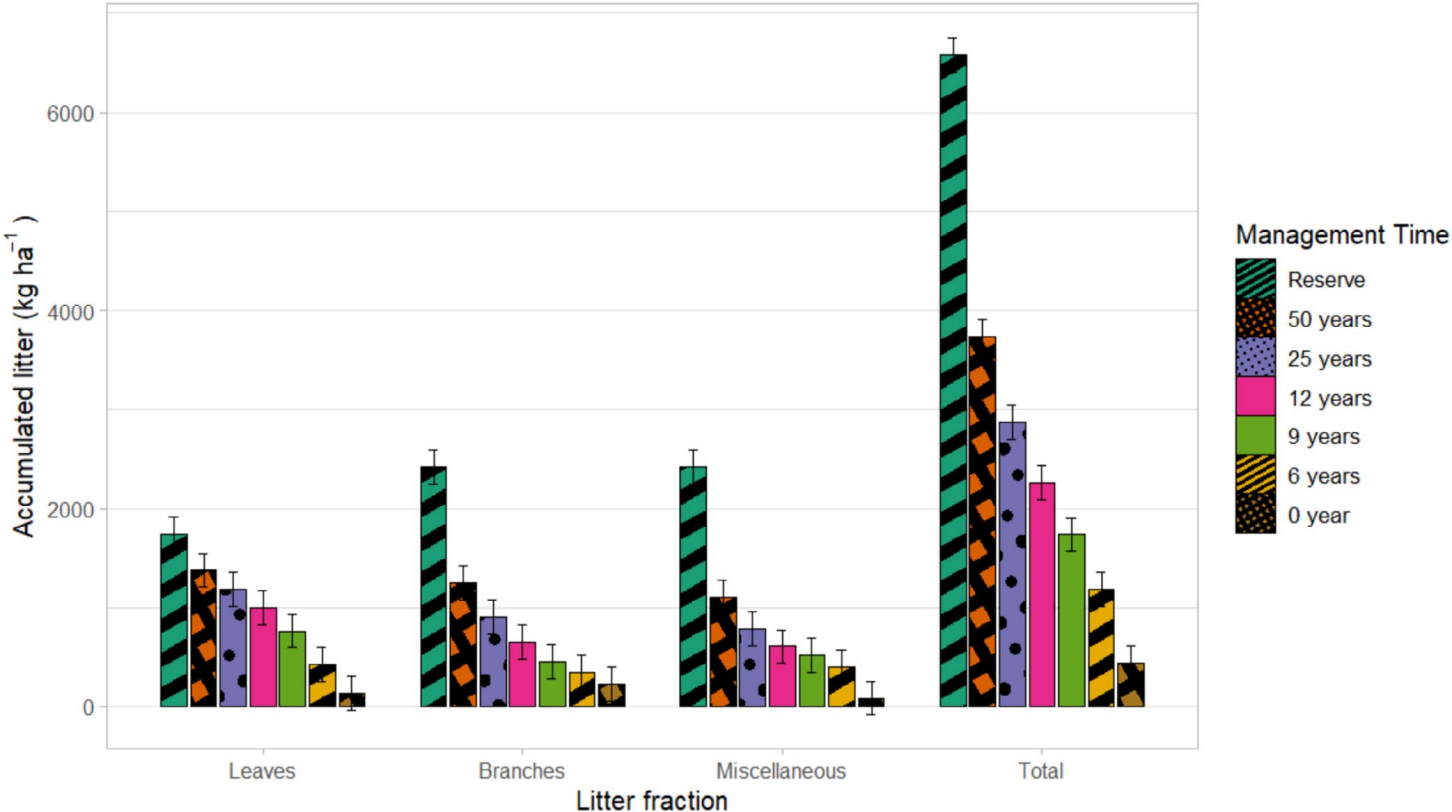
Total accumulated litter of the Caatinga and its fractions at different times of forest management.

The leaf fraction represented the main contribution to the total stock, with a predominance of approximately 60% (Figure 5) in the areas with the greatest coverage. The miscellaneous fraction had the lowest relative values (< 2%), with no statistically significant differences between treatments, although it was more expressive in areas with a predominance of herbaceous plants and sub‐shrubs.

### 3.2 Nutrients in Litter

There was a wide variation in macronutrient content between the litter compartments (leaves, branches and miscellany), reflecting the diversity of species and the different composition of the materials. The leaf and litter fractions had the highest nutrient levels, which is explained by the high content of chlorophyll and associated metabolic compounds (Ohkama‐Ohtsu and Wasaki 2010).

The nitrogen content differed significantly between the litter fractions (*χ* ^2^ Wald (4) = 16.52; *p* = 0.0024). The highest concentration was observed in the miscellaneous fraction, with area 0 having the highest concentration (Table 1). Area 0 differed significantly from the others, especially area R (ΔM = 3.14 g kg^−1^; 95% CI [2.01; 4.27]; *p* < 0.001).

**TABLE 1:** Concentrations of TN, P, and K in the accumulated litter fractions of the Caatinga at different times of forest management.

Areas	Leaves	Twigs	Miscellany
	g kg^−1^		
Total Nitrogen (TN)			
R	14.62 Ba	13.13 Aa	13.77 Ba
50	12.60 Ba	12.42 Aa	15.49 Aa
25	17.25 Aa	15.92 Aa	18.29 Aa
12	16.27 Aa	13.57 Aa	17.70 Aa
9	11.34 Bb	12.07 ABab	15.10 Ba
6	10.93 Ba	10.47 Ba	9.04 Ca
0	10.54 BCa	9.77 Ba	11.07 Ca
Phosphorus (P)			
R	1.65 Aa	1.30 Aa	1.84 Ab
50	1.15 Bb	0.95 Cb	1.70 Ab
25	1.02 Ac	0.96 Ab	1.14 Ad
12	1.95 Aa	1.31 Ba	2.12 Aa
9	1.30 Ab	0.82 Bb	1.41 Ac
6	1.34Ab	0.83 Bb	1.02 Bd
0	0.99 Bc	1.14 Ba	1.42 Ac
Potassium (K)			
R	3.41 Ca	3.45 Aa	3.38 Bb
50	3.87 Ba	3.23 Ac	3.44 Ba
25	3.32 Ca	2.51 Bb	2.50 Cb
12	4.23 Ba	2.59 Bb	4.03 Aa
9	5.07 Aa	2.79 Ab	2.76 Bb
6	4.12 Ba	3.39 Ab	3.25 Bb
0	4.38 Aa	2.39 Bb	2.83 Bb

*Note:* (1) Adjusted means followed by different letters differ from each other by the pairwise multiple comparisons test, based on the scale of the dependent variable (PeS), at a significance level of 5% (*p* < 0.05), as estimated by the GLM model. Uppercase letters indicate significant differences between the different areas for the same fraction, while lowercase letters indicate differences between the different fractions within the same area. Comparisons were made between environments (cutting times) and between materials (litter fractions), with adjustment for standard error and Wald confidence interval.

As for the concentration of nitrogen in the leaf fraction, the highest levels were recorded in the 25‐year‐old (17.25 g kg^−1^) and 12‐year‐old (16.27 g kg^−1^) areas. The 25‐year‐old area showed a statistically significant difference compared to area 0 (ΔM = 6.71 g kg^−1^; 95% CI [5.252; 8.170]; *p* < 0.001).

As with the leaf fraction, the highest nitrogen contents in the twig fraction were found in areas 25 (15.92 g kg^−1^) and 12 (13.57 g kg^−1^). The 25‐year‐old area showed a statistically significant difference, with higher levels compared to area 0 (ΔM = 6.16 g kg^−1^; 95% CI [4.32; 7.99]; *p* < 0.001), area 6 (ΔM = 5.46 g kg^−1^; 95% CI [3.63; 7.28]; *p* < 0.001) and area 9 (ΔM = 3.32 g kg^−1^; 95% CI [1.50; 5.13]; *p* < 0.001).

The miscellanea fraction showed the highest phosphorus levels, especially in area 0, which differed statistically from area 12 (ΔM = 3.80 g kg^−1^; 95% CI [0.17; 7.46]; *p* = 0.032) and area 25 (ΔM = 6.16 g kg^−1^; 95% CI [2.52; 9.79]; *p* < 0.001).

The highest potassium contents were observed in the leaf and miscellaneous fractions, with the 25‐year‐old area showing the highest concentrations, with a statistically significant difference compared to the 0‐year‐old area (ΔM = 7.23 g kg^−1^; 95% CI [3.59; 10.87]; *p* < 0.001). Although differences were observed in relation to the 50‐year‐old (ΔM = 2.80 g kg^−1^; 95% CI [0.83; 6.44]; *p* = 0.377) and 9‐year‐old (ΔM = 3.19 g kg^−1^; 95% CI [0.44; 6.84]; *p* = 0.152) areas, these were not statistically significant. The twig fraction had the lowest potassium content.

Calcium content differed statistically between the areas evaluated (*χ* ^2^ Wald (6) = 2.79; *p* = 0.016), with a significant interaction between environment and litter fraction (*χ* ^2^ Wald (17) = 3.17; *p* < 0.001). The highest concentration was observed in the leaf fraction, especially in the 12‐year‐old (20.65 g kg^−1^) and 25‐year‐old (20.59 g kg^−1^) areas, while area R had the highest overall average considering all fractions (20.43 g kg^−1^).

The 12‐year‐old area differed significantly from area 0 (ΔM = 3.89 g kg^−1^; 95% CI [1.97; 5.81]; *p* < 0.001), and from area 9 (ΔM = 3.39 g kg^−1^; 95% CI [1.50; 5.28]; *p* < 0.001).

The magnesium content differed significantly between the areas evaluated (*χ* ^2^ Wald (6) = 15.28; *p* = 0.018), with statistical variation especially in the miscellaneous fraction. The highest concentration was observed in the 12‐year‐old area (1443 g kg^−1^), which stood out compared to the 0‐year‐old (0.93 g kg^−1^), 6‐year‐old (1028 g kg^−1^) and 9‐year‐old (1.228 g kg^−1^) areas. The difference between the 12‐year area and the 0‐year area (Table 2) was statistically significant (ΔM = 0.36 g kg^−1^; 95% CI [0.23; 0.49]; *p* < 0.001).

**TABLE 2:** Concentrations of Ca, Mg, and S in the total litter fractions of the Caatinga at different times of forest management.

Areas	Leaves	Twigs	Miscellany
	g kg^−1^		
Calcium (Ca)			
R	20.43Aa	20.07 AB	19.89 Aa
50	19.42 Aa	17.24 Ba	17.83 Aa
25	20.59 Aa	17.42 Ba	20.71 A a
12	20.65 Aa	18.78 Bb	17.06 Ab
9	17.26 Bb	22.61 Aa	17.63 Ab
6	17.‐23.10 Bb	20.96 Aa	17.36 Ab
0	16.76 Ba	19.11 ABa	17.91 Aa
Magnesium (Mg)			
R	1.08 ABa	1.16 Aa	0.98 Bb
50	1.23 Aa	1.19 Aa	1.19 Aa
25	0.95 ABb	1.28 Ab	1.37 Aa
12	1.22 Ab	1.38 Ab	1.44 Aa
9	1.02 ABa	0.94 Ba	1.23 Aa
6	0.88 Ba	1.06 Ba	1.03 Ba
0	1.38 Aa	0.65 Cc	0.93 Bb
Sulfur (S)			
R	3.52 Aa	3.22 Ba	2.48 Cb
50	2.15 Bb	3.44 Bb	5.03 Aa
25	2.30 Bc	5.50 Aa	4.63 Ab
12	4.49 Aa	1.37 Cb	3.06 Ba
9	1.73 Bb	4.36 ABa	4.50 Aa
6	2.10 Ba	1.69 Ca	2.60 Ca
0	1.72 Ba	1.23 Ca	2.47 Ca

*Note:* Adjusted means followed by different letters differ from each other by the pairwise multiple comparisons test, based on the scale of the dependent variable (PeS), at a significance level of 5% (*p* < 0.05), as estimated by the GLM model. Uppercase letters indicate significant differences between the different areas for the same fraction, while lowercase letters indicate differences between the different fractions within the same area. Comparisons were made between environments (cutting times) and between materials (litter fractions), with adjustment for standard error and Wald confidence interval.

### 3.3 Total Carbon Stock and TC/TN Ratio

The total carbon content differed significantly between the areas (*χ* ^2^ Wald (6) = 3.89; *p* = 0.0018). The 25‐year‐old (320.56 g kg^−1^) and 9‐year‐old (337.82 g kg^−1^) areas had the highest concentrations, with a statistically significant difference from area 6 (210,14 g kg^−1^). The comparison between the 9‐year‐old area and the 6‐year‐old area revealed a significant average difference (ΔM = 127.68 g kg^−1^; 95% CI [83.70; 171.67]; *p* < 0.001).

As for the accumulated litter fractions, there was no statistically significant difference when considered separately (Table 3).

**TABLE 3:** TC concentration and TC/TN ratio in the accumulated litter fractions of the Caatinga at different times of forest management.

Areas	Leaves	Twigs	Miscellany
	g kg^−1^		
Total Carbon (TC)			
R	371.26 Aa	288.97 Aab	248.74 ABb
50	356.52 Aa	298.57 Aa	290.41 ABa
25	335.80 Aa	319.50 Aa	320.56 Aa
12	345.43 Aa	310.77 Aa	308.67 ABa
9	295.25 Aa	338.82 Aa	337.82 Aa
6	279.20 Aab	308.95 Ab	210.14 Ba
0	376.56 Aa	247.96 Ab	231.38 ABb
TC/TN			
R	−25.30	21.96 B	18.28 A
50	28.76 Aa	24.15 Ba	18.75 Ab
25	19.39 Ba	20.11 Ca	17.650 Aa
12	21.98 Ba	23.06 Ba	17.31 Aa
9	26.09 Aa	28.54 Ba	22.51 Aa
6	25.52 Ba	30.42 Aa	22.74 Aa
0	35.61 Aa	25.68 Bb	20.80 Ab

*Note:* Adjusted means followed by different letters differ from each other by the pairwise multiple comparisons test, based on the scale of the dependent variable (PeS), at a significance level of 5% (*p* < 0.05), as estimated by the GLM model. Uppercase letters indicate significant differences between the different areas for the same fraction, while lowercase letters indicate differences between the different fractions within the same area. Comparisons were made between environments (cutting times) and between materials (litter fractions), with adjustment for standard error and Wald confidence interval.

The TC/TN ratio differed significantly between the environments evaluated (*χ* ^2^ Wald (6) = 10.59; *p* < 0.001), with an overall average of 23.51. The highest mean was observed in the 0‐year‐old area (27.34), significantly higher than the 12‐year‐old (ΔM = 6.67; 95% CI [2.46; 10.87]; *p* < 0.001), 25‐year‐old (ΔM = 8.31; 95% CI [4.11; 12.52]; *p* < 0.001) and reference area (ΔM = 5.37; 95% CI [1.16; 9.58]; *p* = 0.003) areas.

The interaction between environment and material was marginally significant (*F*(17, 84) = 1.610; *p* = 0.059), suggesting that the TC/TN ratio patterns vary between the fractions (leaves, branches and miscellany), although without statistical significance at the 5% level.

The leaf fraction had the highest TC/TN ratios in all environments, especially in the 0‐year area (35.61 g kg^−1^), while the miscellany had the lowest values, especially in the 25‐year (17.65 g kg^−1^) and reference area (18.28 g kg^−1^) areas. The difference between leaves and miscellany in the 0‐year‐old area was statistically significant (ΔM = 14.80 g kg^−1^; 95% CI [11.86; 17.74]; *p* < 0.001).

### 3.4 PCA and Clustering

Principal Component Analysis (PCA) was applied to identify multivariate patterns between structural attributes of the litter (NDF, Hemicellulose, Lignin, Cellulose and FDA). The first two principal components jointly explained 82.7% of the total variability in the data, with PC1 accounting for 48.8% and PC2 for 33.9% (Table 4).

**TABLE 4:** Eigenvalues, explained variance and accumulated variance (%) of the principal components extracted from the PCA.

Component	Eigenvalue variance	Cumulative variance
PC1	2.439	48.8%
PC2	1.694	33.9%
PC3	0.847	16.94%
PC4	0.013	0.27%
PC5	0.004	0.09%

PC1 represented a dominant axis of lignocellulosic recalcitrance, with high negative factor loadings for FDA (−0.606), Cellulose (−0.489) and NDF (−0.484), and a positive contribution from Hemicellulose (0.396). The high representation of the attributes FDA from the square of the cosine (cos^2^) at 0.896, Cellulose (cos^2^ = 0.582) and NDF (cos^2^ = 0.571) confirms the strong association of this axis with the total content of structural fibers resistant to decomposition, indicating greater potential for physical stability and persistence of biomass in environments with negative coordinates on this axis (Table 5).

**TABLE 5:** Square of the cosine (cos^2^) of the variables in the principal components.

Variable	PC1	PC2	PC3	PC4	PC5
NDF	0.571	0.249	0.174	0.006	0.000
Hem	0.383	0.039	0.576	0.002	0.000
Lignin	0.008	0.934	0.056	0.001	0.001
Cellulose	0.582	0.374	0.040	0.001	0.002
FDA	0.896	0.099	0.000	0.004	0.001

PC2 showed an independent gradient, characterized by a strong negative charge for Lignin (−0.742) and a positive charge for Cellulose (0.470), suggesting an axis of contrast between aromatic components and structural carbohydrates. The high quality of Lignin's representation in this component (cos^2^ = 0.934) reinforces its differentiating function, associated with more recalcitrant materials that are less susceptible to microbial decomposition. Hemicellulose, on the other hand, had a marginal contribution in PC2 (cos^2^ = 0.039), denoting its more variable nature.

**FIGURE 6:**
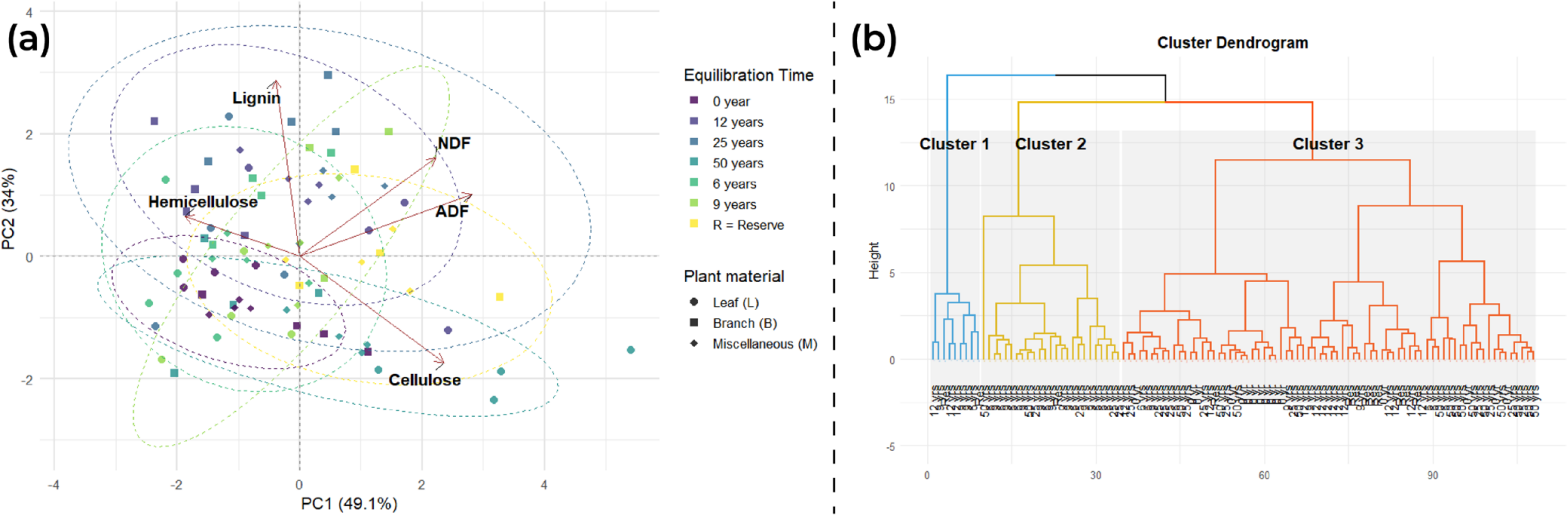
Integrated analysis of the structural composition of the litter. (a) Projection of the Principal Component Analysis (PCA) of the samples onto the plane defined by the first two principal components, considering the attributes NDF, cellulose, lignin, hemicellulose, and FDA; (b) Hierarchical clustering dendrogram based on the lignocellulosic composition of the litter, identifying three functional groups (Clusters 1, 2, and 3) with distinct structural signatures.

The projection of the samples in multivariate space (Figure 6a) shows a distribution consistent with structural patterns. Samples located in regions with negative PC1 coordinates showed greater accumulation of complex fibrous components (FDA, NDF, Cellulose), while those with more positive values were associated with materials with higher relative hemicellulose content and lower lignocellulosic complexity. The distribution along PC2 also highlighted samples with higher lignin contents, isolating them in the lower quadrants.

The hierarchical cluster analysis based on the structural attributes of the litter revealed the formation of three distinct compositional groups (Figure 6b), reflecting specific patterns of lignocellulosic constitution. As observed in the Principal Components Analysis (PCA), Cluster 1 had the highest average recalcitrance index (0.715) and was characterized by high cellulose content (52.2%) and a lower proportion of lignin (15.4%).

Cluster 2 showed intermediate values for the main structural components, with lignin of around 20.3%, cellulose of 30.2%, and a recalcitrance index of 0.623, configuring a balanced composition with moderate recalcitrance.

On the other hand, Cluster 3, with the lowest recalcitrance index (0.356), was marked by the highest lignin content (36.8%) and the lowest cellulose content (20.5%).

Cluster 3, with a high concentration of lignin, also had the highest C/N ratios (27.34 in the area with 0 years of regeneration). In contrast, Cluster 1, with its higher cellulose content and lower C/N (17.65 in the 25‐year‐old area), was associated with a higher turnover rate.

The results of the linear regressions, together with the structural patterns extracted from the PCA and cluster analysis, point to a strong coupling between the structural quality of the litter and its capacity to accumulate nutrients throughout the regeneration chronosequence. The data show that the accumulated biomass of litter is associated with different chemical attributes of the soil, with significant variations between the fractions analyzed. In the Leaves fraction, there was a significant effect of calcium (*β* = 84.830; *p* = 0.013; 95% CI = 20.080–149.580), total nitrogen (*β* = 59.470; *p* = 0.023; 95% CI = 9.030–109.920) and C/N ratio (*β* = −35.580; *p* = 0.017; 95% CI = −64.080 to −7.080), indicating that greater leaf biomass accumulation is positively associated with poorly mobile structural nutrients and negatively related to the recalcitrance index.

**FIGURE 7:**
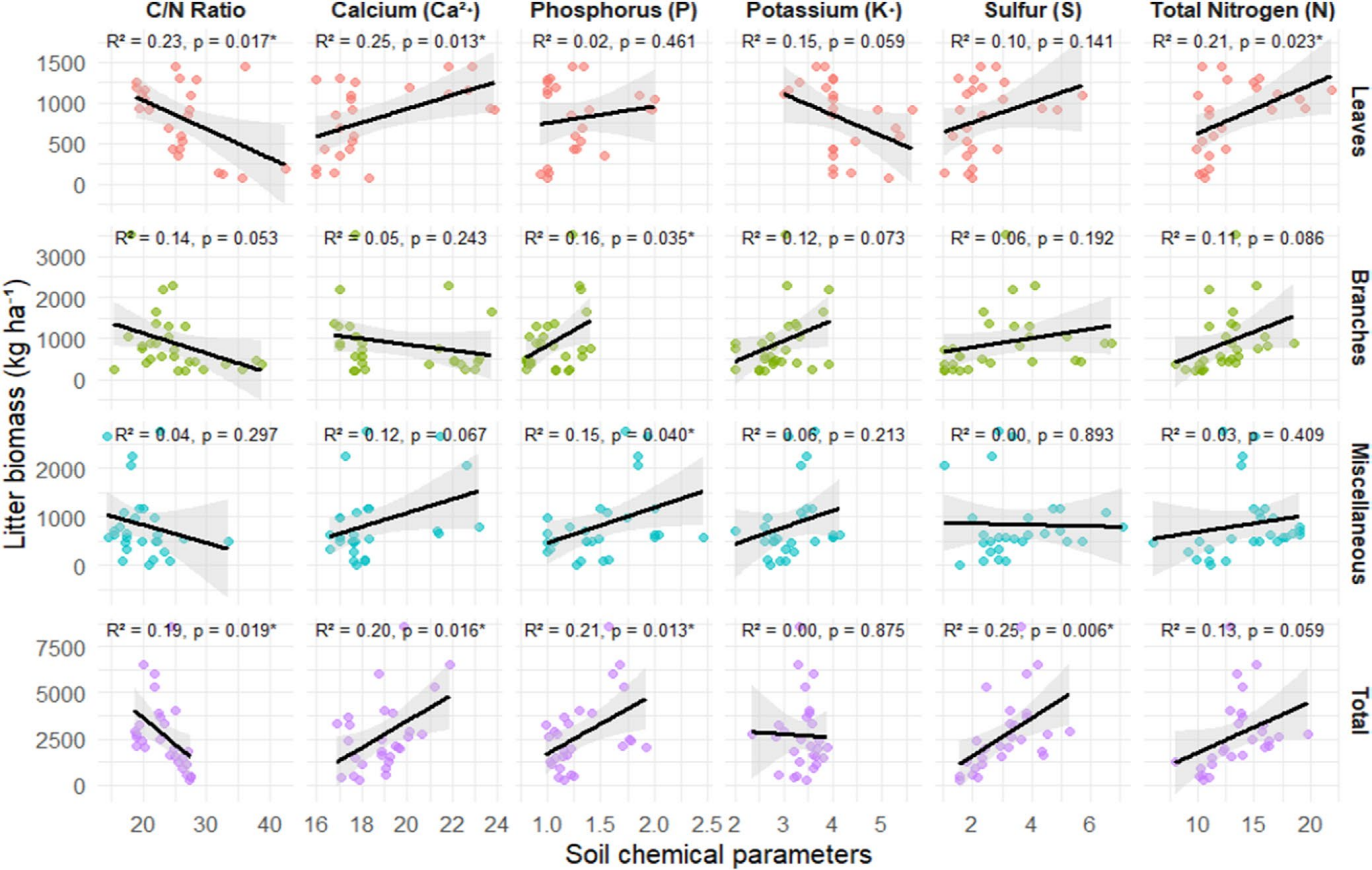
Linear regression models between litter biomass (kg ha^−1^) and soil chemical parameters, stratified by litter fraction (Leaves, Twigs, Miscellaneous and Total). Each facet represents the relationship between a specific chemical variable and accumulated biomass. The regression line (black) is shown with a 95% confidence interval (gray shaded area). The coefficient of determination (*R* ^2^) and *p*‐value are indicated in the top right‐hand corner of each panel. The asterisk (*) indicates statistically significant models (*p* < 0.05).

In the Twigs fraction (Figure 7), biomass showed a significant relationship only with phosphorus (*β* = 1493.380; *p* = 0.035; 95% CI = 110.560–2876.210), which suggests localized retention of more labile nutrients in woody structures under certain successional stages. In the Miscellaneous fraction, both phosphorus (*β* = 728.040; *p* = 0.040; 95% CI = 36.440–1419.650) and calcium (*β* = 141.160; *p* = 0.067, marginal) indicated relevant associations with biomass accumulation, although with less statistical robustness.

Considering the total biomass, the regression indicated positive and statistically significant effects of phosphorus (*β* = 3228.460; *p* = 0.013; 95% CI = 731.030–5725.890), calcium (*β* = 717.630; *p* = 0.016; 95% CI = 143.430–1291.830), sulfur (*β* = 1006.770; *p* = 0.006; 95% CI = 309.770–1703.770) and C/N ratio (*β* = −283.680; *p* = 0.019; 95% CI = −516.400 to −50.960), reflecting the synergistic role between structural nutrients and plant material quality in litter persistence.

## 4 Discussion

### 4.1 Total Litter

The total accumulated litter stocks were higher than those observed by Alves et al. (2017) in hyperxerophilic Caatinga areas under regeneration (5.3 t ha^−1^) and preserved (10.0 t ha^−1^), as well as the 7.0 t ha^−1^ reported by Amorim et al. (2014) in the semi‐arid region. Similarly, Holanda et al. (2017) recorded an annual litter production of 3.79 t ha^−1^ in a Caatinga fragment in Paraíba, predominantly composed of leaf material. Vegetation structure, modulated by regeneration time, directly influenced litter stocks.

This pattern is directly related to the successional trajectory of the vegetation, in which more recently managed areas have a shrub‐herbaceous physiognomy and low tree density, while areas with more regeneration time develop a continuous canopy and a predominance of tree strata.

Nguyen et al. (2024) showed that forests with greater vegetation cover have higher carbon inputs through litter, as a result of greater primary production in structured environments. Similarly, Joshi and Garkoti (2023) observed a significant increase in carbon and nutrient stocks over a forest chronosequence, reinforcing the role of tree structure in biomass accumulation. Gogoi et al. (2020) also pointed out that areas with a longer time since the last disturbance have more closed cover, greater diversity, and higher carbon stocks compared to areas undergoing initial regeneration. In addition, older natural forests tend to accumulate greater amounts of litter which, depending on its decomposition stage, influences microbial community composition and activity, particularly bacteria, thus affecting carbon cycling processes (Chen, Li, et al. 2024).

This predominance is associated with the deciduous phenology of Caatinga vegetation, whose leaf fall is intensified by water stress (Rufino et al. 2024). Holanda et al. (2017) noted that litterfall in the Caatinga coincides with the dry season, reinforcing the link between leaf senescence and rainfall seasonality. Classic and contemporary studies indicate that leaves make up between 60% and 80% of the litter in tropical and seasonal forests, due to their high turnover and shorter time in the canopy, especially under water stress (Bray and Gorham 1964). This predominance also reflects the higher content of photosynthesizing tissues and the influence of deciduous species adapted to the semi‐arid region. This was also observed by Holanda et al. (2017), who reported that the leaf fraction accounted for 70.2% of the total litterfall in a Caatinga fragment in Paraíba.

The twig fraction, possibly influenced by structural senescence and the action of mechanical agents such as wind and rain (Arrais Benício et al. 2023), shows irregular deposition related to episodic events such as prolonged drought, strong wind, or mechanical breakage. Such patterns have been documented by König et al. (2002) and Umaña (2023) in studies of dry forests.

The miscellaneous fraction, although minimal, was more expressive in areas with a predominance of herbaceous plants and sub‐shrubs, possibly due to the anemochoric dispersal of propagules. Holanda et al. (2017) also identified contributions from reproductive structures (18.3%) and miscellaneous material in Caatinga litter, showing the variability in composition across physiognomies and seasonal conditions.

The results observed in the PCA suggest that the content and structural composition of the litter respond to gradients defined by senescence processes, biomass recomposition, and the intrinsic characteristics of the dominant species in different environments. Tonin et al. (2021) demonstrated that the chemical composition of litter varies seasonally and is modulated by the leaf phenology of the dominant species in tropical forests, with structural changes associated with environmental variations and senescence.

As discussed, Cluster 1 represents more easily degradable biomass, while Cluster 3 expresses resistance to decomposition. Similar results were reported by Bonnefoy‐Claudet et al. (2025), reinforcing the inverse relationship between recalcitrance and structural simplicity. Similar patterns have been observed in temperate forests (Zhao et al. 2022) and mangroves (Pradisty et al. 2021), reinforcing the idea that lignocellulosic quality determines decomposition rates and organic matter persistence. More advanced areas tend to accumulate nutrient‐rich and persistent biomass, while recently disturbed areas produce less complex, short‐lived material, reinforcing the idea that restoring soil function requires both biomass and its structural‐nutritional quality (Saha et al. 2023; Zhang et al. 2023; Jiang et al. 2025).

### 4.2 Nutrients in Litter

Species diversity and different nutrient contents are fundamental to the balance of mineral nutrition in ecosystems. Species with a greater cycling capacity contribute to the continuous release of nutrients, avoiding losses through leaching and promoting system stability, as demonstrated by Fontaine et al. (2024), who highlight the importance of plant–soil interactions in synchronizing nutrient supply and demand in biodiverse systems.

These results are in line with Santana (2005), who observed the highest levels in the miscellany (24.863 g kg^−1^) and the lowest in the branches (9.892 g kg^−1^), as well as being similar to those reported by Souto et al. (2009) and higher than those of Barbosa et al. (2019). The nutrient concentrations observed in the present study, particularly in the leaf fraction, are consistent with the patterns reported by Holanda et al. (2017), who found that calcium was the most abundant element, followed by nitrogen and potassium, reflecting the predominance of structural and metabolic components in photosynthetic tissues.

Nitrogen levels in the leaf litter ranged from 10.5 to 17.3 g kg^−1^, and phosphorus reached up to 1.95 g kg^−1^ in areas with more ad Sced regeneration, emphasizing the role of successional stages in nutrient accumulation. These values are comparable to those found by Holanda et al. (2017), who also emphasized the seasonal variability of nutrient deposition in Caatinga litter and the influence of rainfall on cycling dynamics.

This agreement reinforces the idea that nutrient dynamics in semi‐arid ecosystems are strongly governed by vegetation structure, successional processes, and climatic seasonality.

The miscellany, made up of organic fragments, insects, and excreta rich in nitrogen compounds, also contributes to the high N levels (Falcón et al. 2014).

In addition, the predominant presence of legumes in the areas studied, with biological nitrogen fixation (BNF) potential, may have intensified this accumulation (Turan and Yildirim 2021). The higher concentration of nitrogen in the leaves can be attributed to its presence in chloroplasts and in essential enzymes such as Rubisco, which play fundamental roles in photosynthesis and cellular respiration, as discussed in the context of biological strategies for soil regeneration and plant metabolic efficiency (Uphoff and Thies 2023).

The composition of the miscellany, made up of organic fragments, insect remains, and excreta rich in nitrogen compounds, also contributes to the high nitrogen levels, as highlighted by Huntley (2023) when addressing the functioning of tropical ecosystems and the dynamics of organic matter. It is also possible that the higher nitrogen contents observed in the miscellaneous fraction may be associated with the presence of N_2_‐fixing legumes, which are common in intermediate stages of succession in the Caatinga, such as species of the genera *Mimosa*, *Piptadenia*, and *Calliandra* (Alamu et al. 2023; Rojas‐Jimenez et al. 2024).

The incorporation of root nodules and leaf material from these species into the litter contributes to enriching the N content, especially in the more heterogeneous fractions, such as the miscellany, which concentrates floral remains, reproductive structures, and woody fragments with a longer residence time (Bonatelli et al. 2021). In addition, the higher N content may indicate greater local microbial activity, favored by edaphic or microclimatic conditions in areas undergoing advanced regeneration (Camelo et al. 2021).

The concentration of phosphorus in leaves is related to its role in photosynthesis and cell biosynthesis, especially due to its presence in phospholipids and coenzymes (Jordan 1985; Havlin et al. 1990). This accumulation may be associated with the presence of animal excreta, rich in phosphate compounds, as discussed by Poirier et al. (2022), which also contribute to the entry of phosphorus into the litter. The soil in the areas studied, characterized by low phosphorus fixation, favors the cycling of the nutrient and its absorption by plants, especially in association with mycorrhizal fungi that optimize P availability even under limiting conditions (Brar et al. 2024).

Potassium, in turn, is a mobile, non‐structural element that is redistributed from leaves to developing tissues. Its function includes enzyme activation, controlling the transport of water and nutrients, and maintaining cell turgidity (Sardans and Peñuelas 2021; Cornut et al. 2021). The highest magnesium values were observed in the leaf and miscellany fractions, which reinforces its metabolic and structural importance, since magnesium is essential for photosynthesis, acting at the center of the chlorophyll molecule and as an enzyme cofactor (Shaul 2002).

These results are in line with White and Broadley (2003), who attribute the accumulation of calcium to its low mobility and structural function in the middle lamella of cells. The values found were higher than those reported by Amorim et al. (2014) for branches and litter, and similar to those of Mlambo and Nyathi (2008) for tropical savannas in Zimbabwe.

In particular, the dominance of negative charges for FDA and NDF in PC1 indicates a greater presence of plant tissues that are difficult to degrade, associated with slower cycles of decomposition and nutrient release (Yi et al. 2023; Wang et al. 2024). Clusters with high lignin (Cluster 3) and high C/N ratios operate as a biochemical mechanism that inhibits microbial decomposition, as lignin blocks hydrolytic enzymes and reduces nitrogen availability (Cuchietti et al. 2014). Cluster 1, even in early succession, maintains lower C/N ratios and facilitates nutrient cycling. Conversely, phosphorus, calcium, and sulfur promote biomass retention, especially in persistent fractions (Meng et al. 2022). The correspondence between the clusters, PCA axes, and regression models confirms the existence of distinct functional profiles across the regeneration chronosequence, with implications for nutrient cycling and soil resilience.

### 4.3 Total Carbon Stock and TC/TN Ratio

The results reinforce that the amount of carbon stored in the soil under natural vegetation reflects a dynamic balance between the addition of plant and animal biomass and the loss through decomposition and mineralization, as demonstrated by Wang et al. (2025) in forest environments sensitive to ecological variation. As forests mature, the accumulation of carbon in the soil and biomass tends to increase, particularly in older successional stages, as shown by Cheng et al. (2025), who reported a continuous increase in soil organic carbon with forest age. Similar findings were observed by Arunrat et al. (2023) in rotational shifting cultivation systems in northern Thailand, where older fallow fields (12 years) exhibited higher stocks of total soil carbon (TC) and total nitrogen (TN) compared to younger fallows (6 years). In the present study, the same trend was observed: older fallow areas showed higher TC and TN, suggesting a progressive restoration of soil quality with increasing fallow duration.

As forests mature, the accumulation of carbon in the soil and biomass tends to increase, being more expressive in more advanced successional stages, as demonstrated by Cheng et al. (2025), who observed a continuous increase in organic carbon with advancing forest age.

The high TC/TN values recorded in this study reflect greater biomass retention and low decomposition rates, a pattern also identified by Lu et al. (2021) in areas with slow litter degradation and high root production, confirming the different ecological conditions of the areas analyzed.

These findings indicate that early‐stage regenerating areas contain organic matter of lower nutritional value, with greater nitrogen immobilization and slower decomposition, compared to more mature areas of forest succession (Welker et al. 2024).

The TC/TN ratio is widely recognized as a key variable in studies on litter decomposition. In addition, this ratio is used as an indicator of the nutritional quality of the material, with values higher than 25 indicating low nitrogen availability and, consequently, a slower rate of decomposition. This pattern was confirmed by Poeplau et al. (2023), who observed that residues with a higher TC/TN ratio promote lower carbon mineralization and greater nitrogen immobilization, reflecting lower organic matter quality.

In this sense, the results showed high TC/TN ratios in the litter (> 25), especially in recently cut areas (0 years) and in the leaf fraction (35.6), indicating low nutritional quality and a potential reduction in decomposition rates. This pattern corroborates studies in dry ecosystems that associate high TC/TN ratios with soil degradation processes. Berthrong et al. (2012) observed that conversions from native vegetation to managed systems reduce organic nitrogen stocks, raising the TC/TN ratio and compromising nutrient cycling—a direct parallel to our post clear‐cutting scenario.

Additionally, Powers et al. (2015) found that TC/TN ratios > 25 reduce microbial activity by 30% to 50% and compromise nutrient cycling after anthropogenic disturbances. The combination of high litter recalcitrance (Cluster 3 with 36.8% lignin) and TC/TN ratios may suggest that clear‐cutting induces a state of low edaphic resilience, where N immobilization prolongs ecosystem recovery.

The values observed in this study indicate that the low nutritional quality of the litter may be a factor limiting decomposition, especially in the branches deposited on the soil surface. This may also reflect greater efficiency on the part of the vegetation in conserving nitrogen in its living tissues, compared to other tropical forest ecosystems (Swift 1996).

Clusters with high lignin (Cluster 3) and high TC/TN ratios operate as a biochemical mechanism that inhibits microbial decomposition, as lignin blocks hydrolytic enzymes and reduces nitrogen availability (Cuchietti et al. 2014). Cluster 1, even in early succession, maintains lower TC/TN ratios and facilitates nutrient cycling. The negative effect of the TC/TN ratio on biomass accumulation (observed in regression) confirms the findings of Duan et al. (2025), where more recalcitrant materials decompose slowly, resulting in lower standing biomass.

## 5 Conclusions

This study demonstrated that clear‐cutting in hyperxerophilous Caatinga substantially alters litter stock accumulation and compromises the efficiency of biogeochemical cycling, thereby reducing ecosystem functional resilience and requiring long regeneration periods to restore litter‐mediated ecosystem services. Nutrient stocks, particularly N and P, were lower in recently disturbed areas, with carbon‐to‐nitrogen (TC/TN) ratios exceeding 35—especially in the leaf litter fraction. Although nutrient stocks increased progressively with regeneration time, the 50‐year site had not yet reached the levels observed in native vegetation, indicating that natural regeneration remains incomplete even after decades.

Multivariate and univariate analyses revealed that litter biomass accumulation is functionally associated with soil chemical attributes—especially phosphorus, calcium, and sulfur—while high TC/TN ratios negatively affect litter accumulation. Enrichment planting with species exhibiting low TC/TN ratios, along with green manuring practices and biodiversity restoration, may accelerate nutrient cycling, improve litter quality, and enhance ecosystem resilience, particularly in the face of climate change.

## Author Contributions

**Renisson Neponuceno de Araújo Filho:** conceptualization (equal), formal analysis (equal), project administration (equal). **Maria Betânia Galvão dos Santos Freire:** conceptualization (equal), formal analysis (equal), methodology (equal), resources (equal). **Fernando José Freire:** conceptualization (equal), data curation (equal), funding acquisition (equal), writing – original draft (equal). **Rinaldo Luiz Caraciolo Ferreira:** supervision (equal), writing – original draft (equal). **Ludmilla Morais Pereira:** writing – original draft (equal). **Luiz Diego Vidal Santos:** formal analysis (equal), resources (equal), software (equal).

## Conflicts of Interest

The authors declare no conflicts of interest.

## Data Availability

All data supporting the results and conclusions presented in this manuscript are available at the following doi: https://doi.org/10.17605/OSF.IO/B4S9J.
